# Presence of Functional Neurotrophin TrkB Receptors in the Rat Superior Cervical Ganglion

**DOI:** 10.3389/fphys.2017.00474

**Published:** 2017-07-11

**Authors:** Pablo Valle-Leija, Angeles Cancino-Rodezno, Berardo M. Sánchez-Tafolla, Erwin Arias, Diana Elinos, Jessica Feria, María E. Zetina, Miguel A. Morales, Fredy Cifuentes

**Affiliations:** ^1^Departamento de Biología Celular y Fisiología, Instituto de Investigaciones Biomédicas, Universidad Nacional Autónoma de México Mexico City, Mexico; ^2^Departamento de Biología Celular, Facultad de Ciencias, Universidad Nacional Autónoma de México Mexico City, Mexico

**Keywords:** neurotrophin receptors, NGF, BDNF, signaling pathways, sympathetic neurons, synaptic plasticity

## Abstract

Sympathetic neurons express the neurotrophin receptors TrkA, p75NTR, and a non-functional truncated TrkB isoform (TrkB-Tc), but are not thought to express a functional full-length TrkB receptor (TrkB-Fl). We, and others, have demonstrated that nerve growth factor (NGF) and brain derived neurotrophic factor (BDNF) modulate synaptic transmission and synaptic plasticity in neurons of the superior cervical ganglion (SCG) of the rat. To clarify whether TrkB is expressed in sympathetic ganglia and contributes to the effects of BDNF upon sympathetic function, we characterized the presence and activity of the neurotrophin receptors expressed in the adult SCG compared with their presence in neonatal and cultured sympathetic neurons. Here, we expand our previous study regarding the immunodetection of neurotrophin receptors. Immunohistochemical analysis revealed that 19% of adult ganglionic neurons expressed TrkB-Fl immunoreactivity (IR), 82% expressed TrkA-IR, and 51% expressed p75NTR-IR; TrkB-Tc would be expressed in 36% of neurons. In addition, using Western-blotting and reverse transcriptase polymerase chain reaction (RT-PCR) analyses, we confirmed the expression of TrkB-Fl and TrkB-Tc protein and mRNA transcripts in adult SCG. Neonatal neurons expressed significantly more TrkA-IR and TrkB-Fl-IR than p75NTR-IR. Finally, the application of neurotrophin, and high frequency stimulation, induced the activation of Trk receptors and the downstream PI3-kinase (phosphatidyl inositol-3-kinase) signaling pathway, thus evoking the phosphorylation of Trk and Akt. These results demonstrate that SCG neurons express functional TrkA and TrkB-Fl receptors, which may contribute to the differential modulation of synaptic transmission and long-term synaptic plasticity.

## Introduction

Neurotrophins carry out multiple functions in the central and peripheral nervous system, including survival, growth, differentiation, and synaptogenesis (Lu and Figurov, 1997). In addition, neurotrophic factors, such as nerve growth factor (NGF) and brain-derived neurotrophic factor (BDNF), acutely regulate synaptic transmission and plasticity (Lu et al., 2004). Neurotrophins bind to, and activate, two types of membrane receptors: the low-affinity p75 neurotrophin receptor (p75NTR) and the tropomyosin-related kinase (Trk) receptor. The Trk receptors, TrkA, TrkB, and TrkC, selectively bind to a specific neurotrophin, whereas p75NTR binds to all neurotrophins (Chao, 2003; Huang and Reichardt, 2003; Lu et al., 2004). Two TrkB receptor isoforms are expressed in the central nervous system (Yan et al., 1997), a truncated 90 kD non-functional isoform (TrkB-Tc) and the full-length 140 kDa receptor (TrkB-Fl), which contains the tyrosine kinase catalytic domain.

In terms of the peripheral nervous system, the current assumption indicates that TrkB-Fl receptors are not expressed in the rat superior cervical ganglion (SCG). This premise is based upon mRNA expression studies, which showed a null or minimal occurrence of the functional full *trkB* isoform in rat SCG (Dixon and McKinnon, 1994; Wetmore and Olson, 1995). However, Ehrhard and Otten (1994) detected the presence of both full and truncated *trkB* mRNA isoforms in rat sympathetic ganglia.

A substantial number of studies have investigated the function of neurotrophins (Nts) in a neuronal-cardiac myocyte co-culture system (Lockhart et al., 1997; Yang et al., 2002). In these studies, the modulation of neuronal firing patterns, and the facilitation of cholinergic transmission, were mainly attributed to the activation of TrkA or p75NTR receptors by NGF, or to the activation of p75NTR by BDNF (Yang et al., 2002; Luther and Birren, 2009; Luther et al., 2013). In these earlier studies, the potential signaling role of TrkB-Fl on sympathetic neuronal function was excluded as a possibility. However, the presence of TrkB-Fl in the SCG cannot be completely ruled out because it has not been fully addressed.

We have been studying the cellular mechanisms underlying long-term potentiation (LTP) in the SCG of adult rats in an *ex vivo* preparation (reviewed by Cifuentes et al., 2013). It is expected that ganglionic LTP enhances tonic efferent impulses to targets, which would modify the normal function of a diverse range of organs, including the heart, blood vessels, and glands. Thus, numerous lines of evidence have associated the expression of ganglionic LTP to the development or aggravation of hypertension in animal models (Alkadhi et al., 2001; Gerges et al., 2002). Recently, we considered whether some of the effects of neurotrophins upon neuronal excitability which are observed in cultured neurons would also occur in the whole SCG and consequently affect synaptic plasticity, especially LTP. Our studies revealed that BDNF increases LTP, while NGF exerts a dual effect in which it increases LTP at high concentration and decreases it at low concentration (Arias et al., 2014). Based upon pharmacological studies, we postulated that the increase in LTP is mediated by p75NTR, while the reduction of LTP was attributable to TrkA (Arias et al., 2014). These opposing effects could be expected given the differential signaling mechanisms of each Nts receptor. However, we could not rule out the participation of TrkB because, besides the presence of TrkA and p75, we detected the presence of some TrkB-IR sympathetic neurons in the rat SCG (Arias et al., 2014); accordingly, TrkB activation could represent an alternative source of the LTP enhancement induced by BDNF. This possibility would indicate that TrkA and TrkB, which are known to activate similar signaling cascades, produce opposite effects, as reported elsewhere (Scott and Ramer, 2010). Given the novelty of this previous finding, we decided to characterize the expression of neurotrophin receptors in the SCG much more comprehensively.

In the present work, we used a range of complementary techniques, including immunohistochemistry (IHC), Western blot analysis, and RT-PCR, to characterize the presence of TrkB (full and truncated isoforms), TrkA, and p75NTR in the SCG. Furthermore, the detection of phospho-Trk-IR and phospho-Akt-IR was used to determine whether the Trk receptors were activated by specific stimuli. The expression of Nts receptors was also analyzed in neonatal SCG, both *in vitro* and in culture. Our results demonstrate that ganglionic sympathetic neurons express functional TrkA and TrkB-Fl receptors, which may contribute to the differential modulation of synaptic transmission, firing patterns, and long-term synaptic plasticity. A preliminary report has appeared elsewhere in Abstract form (Valle et al., 2014).

## Materials and methods

### Animals

Experiments were carried out using Wistar rats: either adult males (230–280 g; 8–10 weeks of age) or newborn rats (4–6 days of age). All protocols were approved by the Committee for the Care and Use of Animals in the Laboratory of the Biomedical Research Institute (National Autonomous University of Mexico). All experimental procedures were performed in accordance with the Ethical Guidelines for the Care and Use of Laboratory Animals from the National Academy of Sciences of the United States, and were approved by our Institutional Committee.

### Immunohistochemistry

For immunohistochemistry (IHC) studies, adult and newborn rats were deeply anesthetized with sodium pentobarbital (125 mg/kg i.p.), and then transcardially perfused with ice-cold phosphate buffer solution (0.01 M PBS, pH 7.4) followed by ice-cold fixative solution (2% paraformaldehyde, 0.18% picric acid in 0.1 M PBS, pH 7.4). The SCG was desheathed, post-fixed with paraformaldehyde and cryoprotected in a 30% sucrose solution. Immunohistochemistry procedures were the same for the entire ganglia and cultured neurons (with the exception of Triton X-100 for the latter). For ganglia, tissue was fixed, cryoprotected, and then transverse sections (14-μm thick) were prepared in a cryostat at −20°C. Sections were blocked with a solution containing 5% normal donkey serum, 5% bovine serum albumin, and the permeabilizing agent, 0.3% Triton X-100, for 1 h. For neurons in culture, cells were fixed with 4% paraformaldehyde for 10 min, rinsed with PBS and blocked for 30 min in 10% normal donkey serum. To study the localization of NtR in the SCG, tissue sections and cultured ganglionic neurons were incubated with the following primary antibodies (diluted in blocking solution) for 16 h at room temperature: rabbit polyclonal anti-TrkB-ID (Santa Cruz Biotechnology, Dallas, TX, USA. Cat# sc-12, 1:500 dilution), anti-TrB-ED (Santa Cruz Biotechnology, Cat# sc-8316, 1:500 dilution), rabbit polyclonal anti-trkA (Abcam, Cambridge, MA, USA. Cat# ab8871, 1:200 dilution), and goat polyclonal anti-p75 (Santa Cruz Biotechnology, Cat# sc-6188, 1:200 dilution). Treatment with the primary antibody was followed by PBS-Tx washes and incubation with an appropriate fluorescent secondary antibodies (in PBS-Tx) for 2 h: either donkey anti-rabbit Alexa Fluor 594 (Jackson ImmunoResearch Labs, West Grove, PA, USA. Cat# 711-585-152, 1:400 dilution) or donkey anti-goat Alexa Fluor 488 (Jackson ImmunoResearch Labs, Cat# 705-546-147, 1:200 dilution), as appropriate.

In order to analyze Trk activation, we determined whether Trk and Akt were phosphorylated in response to certain treatments (see below). First, SCG was fixed with paraformaldehyde (2% paraformaldehyde, 0.18% picric acid in 0.1 M PBS, pH 7.4), processed as described above, and exposed to rabbit monoclonal anti-phospho-TrkA (Tyr678/675)/TrkB (Tyr706/707) and rabbit anti-phospho-Akt (Ser473), both acquired from Cell Signaling Technology (Danvers, MA, USA. Cat# 4621, 1:200 dilution; and Cat# 9271, 1:200 dilution). Appropriate conjugated anti-rabbit secondary antibodies were used to visualize positive antibody binding within the cells. For controls, some tissue sections, and fixed neurons, were processed through all of the incubation steps but without primary antibodies, or pre-adsorbed overnight at room temperature with a 10-fold molar excess of their corresponding control antigens. Finally, sections and neurons were covered with coverslips using DAKO mounting medium to preserve fluorescence and finally examined with an epifluorescence microscope (Nikon Eclipse E600) or with a Nikon A1R confocal microscope.

### Co-cultures of neurons from SCG and ventricular myocytes

Most of the previous functional studies in this field have been performed in co-cultures of neonatal sympathetic neurons and cardiac ventricular myocytes (Yang et al., 2002; Luther and Birren, 2009). For our study, we used the procedures described by Lockhart et al. (1997). Firstly fresh sympathetic neurons (7,500–15,000 neurons) were isolated from newborn Wistar rats on postnatal day 4–6 (P4–6) and plated with cardiac myocytes (50,000 myocytes) obtained from the same animals. The cells were cultured in L15- CO_2_ medium (Hawrot and Patterson, 1979) plus 10% fetal bovine serum, 6 mg/ml dextrose, 2 mM glutamine (Whittiker), in the presence of NGF (5 ng/mL 2.5S NGF; Upstate Biotech, Lake Placid, NY), as described by Lockhart et al. (1997). After 1 day in culture, 1 μM cytosine arabinofuranoside (AraC; Sigma, St. Louis, MO) was added to the dishes to prevent cell division. Cultures were grown on glass-bottomed dishes (MatTek, Ashland, MA), maintained in culture for 3 or 20 days *in vitro* (DIV).

### Protein isolation and semi-quantitative western blot analysis

Superior cervical ganglia were dissected, immediately frozen in liquid nitrogen, and homogenized with a Polytron homogenizer (Kinematica, New York, NY, USA) in 0.25 mL of ice-cold lysis buffer (in mM: 150 NaCl, 1 EDTA, 0.5 DTT, 20 Tris, 0.5% Triton X-100, pH 8.0) containing 1% SDS and 1X proteinase inhibitor cocktail (Roche Applied Sciences, Indianapolis, IN, USA). Following centrifugation at 1,500 × g, protein concentration was determined from the supernatant by a BCA protein assay (Thermo Scientific, Waltham, MA, USA). Sample preparation was performed in accordance with the Laemmli method (Laemmli, 1970). SDS-PAGE (12.5% resolving gel) was used to separate sample proteins, and a protein standard was used to confirm molecular mass. Protein was then transferred onto a Immobilon-P transfer membrane (Merck Millipore, Billerica, MA, USA), protein transfer was confirmed by Ponceau S staining, and the membrane was blocked with 5% bovine serum albumin (BSA). The following primary antibodies were used for Western blotting: rabbit polyclonal anti-TrkB-ED (Santa Cruz Biotechnology, Cat# sc-8316, 1:500 dilution), rabbit polyclonal anti-TrkA (Abcam, Cat# ab8871, 1:200 dilution), and goat polyclonal anti-p75 (Santa Cruz Biotechnology, Cat# sc-6188, 1:200 dilution). Appropriate secondary HRP-conjugated antibodies were used to visualize proteins as a chemiluminescent HRP substrate (Millipore, Billerica, MA, USA). Proteins of interest were detected using photographic films, which were scanned using the ImageQuant LAS4000 Imaging System (GE Healthcare, Piscataway, NJ, USA). Each band shown herein is from a representative experiment of at least four experimental repetitions.

### RNA isolation and reverse-transcriptase polymerase chain reaction assay

Expression of *trkB-Fl* and *trkB-Tc* mRNA in the SCG was analyzed by reverse-transcriptase polymerase chain reaction (RT-PCR). Total RNA from rat SCG samples was isolated with TRIzol reagent according to the manufacturer's instructions (Life Technologies, Carlsbad, CA, USA). For each reverse transcription reaction, 1.3 μg of total RNA, 500 ng of random primers (Promega, Madison, WI, USA), and High-Capacity Reverse Transcription Kit (Thermo Fisher Scientific, Waltham, MA, USA) were used according to the manufacturer's instructions. cDNA amplification reactions were performed with a PCR Master Mix (Promega, Madison, WI, USA) with specific oligonucleotides designed to amplify a fragment of 245 bp (nt 2601–2845) from *trkB-Fl* and 143 bp (nt 1955–2097) from *trkB-Tc* polypeptide genes, based on the transcriptome of *Rattus norvegicus* (Kondo et al., 2010; NCBI, Reference Sequence: NM_012731.2 and NM_001163168.2). A fragment of 301 bp (nt 176–476) from the GAPDH gene, based on the transcriptome of *Rattus norvegicus* (NCBI, Reference Sequence: NM_017008.4), was also amplified using specific primers (Table 1) to act as a RT-PCR normalization control. To amplify *trkB-Fl* and *trkB-Tc* fragments, we used the following profile: an initial denaturing step at 94°C for 2 min, followed by 40 cycles at 94°C for 30 s, 61°C for 30 s, and 72°C for 30 s, with a final extension period at 72°C for 7 min. To amplify GAPDH fragments, we used the following profile: an initial denaturing step at 94°C for 2 min, followed by 30 cycles at 94°C for 30 s, 43°C for 30 s, and 72°C for 30 s, with a final extension period at 72°C for 7 min. PCR products were separated in a 1.5% agarose gel stained with ethidium bromide and digitized using a Typhoon FLA 9500 laser scanner (GE Healthcare Bio-Sciences Corp., Piscataway, NJ, USA). At least five independent biological samples were used for RT-PCR. Negative controls were included for each reaction and represented reactions without reverse-transcribed mRNA or reaction mixes without templates.

**Table 1:** Primers used for PCR amplification.

**Target cDNA**	**Primers (5′-3′) forward/reverse**	**Amplicon position**	**NCBI Reference Sequence**	**Amplicon size (bp)**
TrkB-FL	TGACGCAGTCGCAGATGCTG/TTTCCTGTACATGATGCTCTCTGG	nt 2601-2845	NM_012731.2	245
trkB-Tc	CGGGAGCATCTCTCGGTCT/AGGGGGATCTTATGAAACAAA	nt 1955-2097	NM_001163168.2	143
GAPDH	CCTTCATTGACCTCAACTAC/TTCACACCCATCACAAAC	nt 176-476	NM_017008.4	301

### Electrophysiological studies

The detection of phospho-Trk-IR and phospho-Akt-IR was used to determine whether the Trk receptors were activated by specific stimuli. We used our routine electrophysiological *in vitro* preparation (Vargas et al., 2007; Arias et al., 2014) in which the ganglion being analyzed was trimmed to increase accessibility to the Nts (Arias et al., 2014). In brief, rats were anesthetized with xylazine (10 mg/kg i.m.) and ketamine (90 mg/kg i.p.), and ganglia were rapidly excised and carefully de-sheathed. The preganglionic and post-ganglionic nerve roots were trimmed to a length of 3–5 mm, and the ganglia were transferred to a recording chamber and bathed with oxygenated (95% O_2_, 5% CO_2_) Krebs-Ringer solution, pH 7.4. For recording and stimulation, the cervical sympathetic trunk (preganglionic), and the internal carotid nerve (post-ganglionic), were pulled into glass suction electrodes to maintain a seal during stimulation and recording. We investigated for potential Trk and Akt phosphorylation in the following conditions: control (trimmed ganglia), high frequency stimulation (tetanic train 40 Hz, 3 s, LTP inductor), BDNF or NGF (200 ng/ml), train plus BDNF (200 ng/ml), and train plus NGF (200 ng/ml). At the end of the assay, the ganglia were fixed by immersion in ice-cold fixative solution and processed for immunohistochemistry, as described above.

### Imaging and cell counting

For cell counting we used a standard procedure which was described previously (Elinos et al., 2016). Briefly, each ganglion was longitudinally sectioned throughout the entire length of its mediolateral axis (~800 μm) to produce 40–50 slices. We sampled the tissue by collecting at least five slices at three depths (140–210, 350–420, and 560–630 μm from the edge of the ganglion) and sorted the slices onto different slides. Sections were photographed with epifluorescence microscopy on a Nikon Eclipse E600 microscope (Tokyo, Japan) with a 20x objective using a high-resolution interline CCD camera (CoolSnap cf camera, Photometrics, Tucson, AZ) and appropriate software; within an experiment all images were acquired under the same exposure conditions. Using an image analysis system (MetaMorph Microscopy Automation and Image Analysis Software, v. 7.5.6, Molecular Devices, Sunnyvale, CA, USA), we removed out of focus blur by means of deconvolution software functions. We then identified specific immunofluorescence labels of immunopositive cells by selecting fluorescent labels that surpasssed negative staining background levels (i.e., > background mean + 2 standard deviations). A different threshold was set to select immunopositive neurons for each marker and each image. In this way, we determined the mean intensity of fluorescence for each neuron. We used DAPI to differentiate between neurons and glia, using the fact that neurons and their nuclei are typically larger than those of glial cells. The proportion (%) of the total number of neurotrophin receptor immunoreactive cells relative to the total number of neurons was used for statistical analysis. The total number of neurons was approximately the same in all explored conditions.

### Statistical analysis

Data are expressed herein as the mean ± standard error of the mean (SEM). Comparison between two groups was performed with the Student's *t*-test, and for multiple groups with one-way analysis of variance **(**ANOVA) followed by Sidak's multiple comparison test; *p* < 0.05 was considered statistically significant.

## Results

### Expression of TrkA, TrkB-Fl, and p75 neurotrophin receptors in the adult SCG

**Figure 1:**
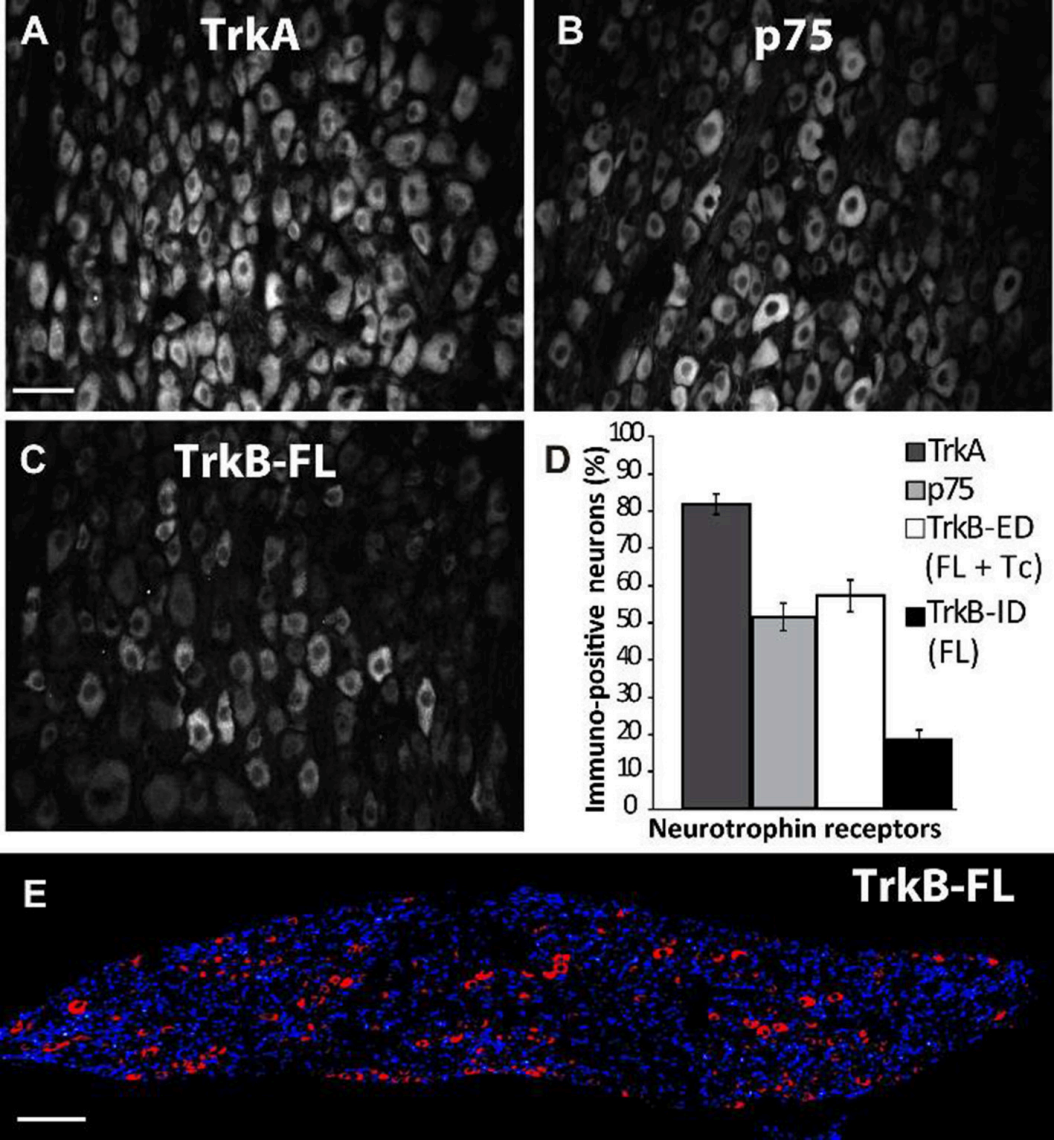
Differential expression of TrkA, p75NTR, and TrkB in rat superior cervical ganglion (SCG). Examples of sympathetic neurons from adult SCG which were immunolabeled against TrkA **(A)**, p75NTR **(B)**, and TrkB-Fl **(C,E)**. Sampling across the entire SCG resulted in different proportions (%) of immunopositive neurons for neurotrophin receptors **(D)**: 83% of the total neuronal population expressed TrkA while 52% expressed p75NTR. An antibody specific for Trk-Fl (that recognizes an intracellular domain) labeled 19% of ganglionic neurons **(C,E)**, whereas another antibody directed against an extracellular domain (ED) common for both TrkB isoforms, full (Fl) and truncated (Tc), stained 55% of neurons. Scale bar = 50 μm **(A–C)**, *n* = 5. **(E)** A transversal slice of the SCG showing DAPI staining (blue) and TrkB-Fl expression (red) indicating that TrkB-Fl is evenly expressed in the SCG. Scale bar = 200 μm.

**Figure 2:**
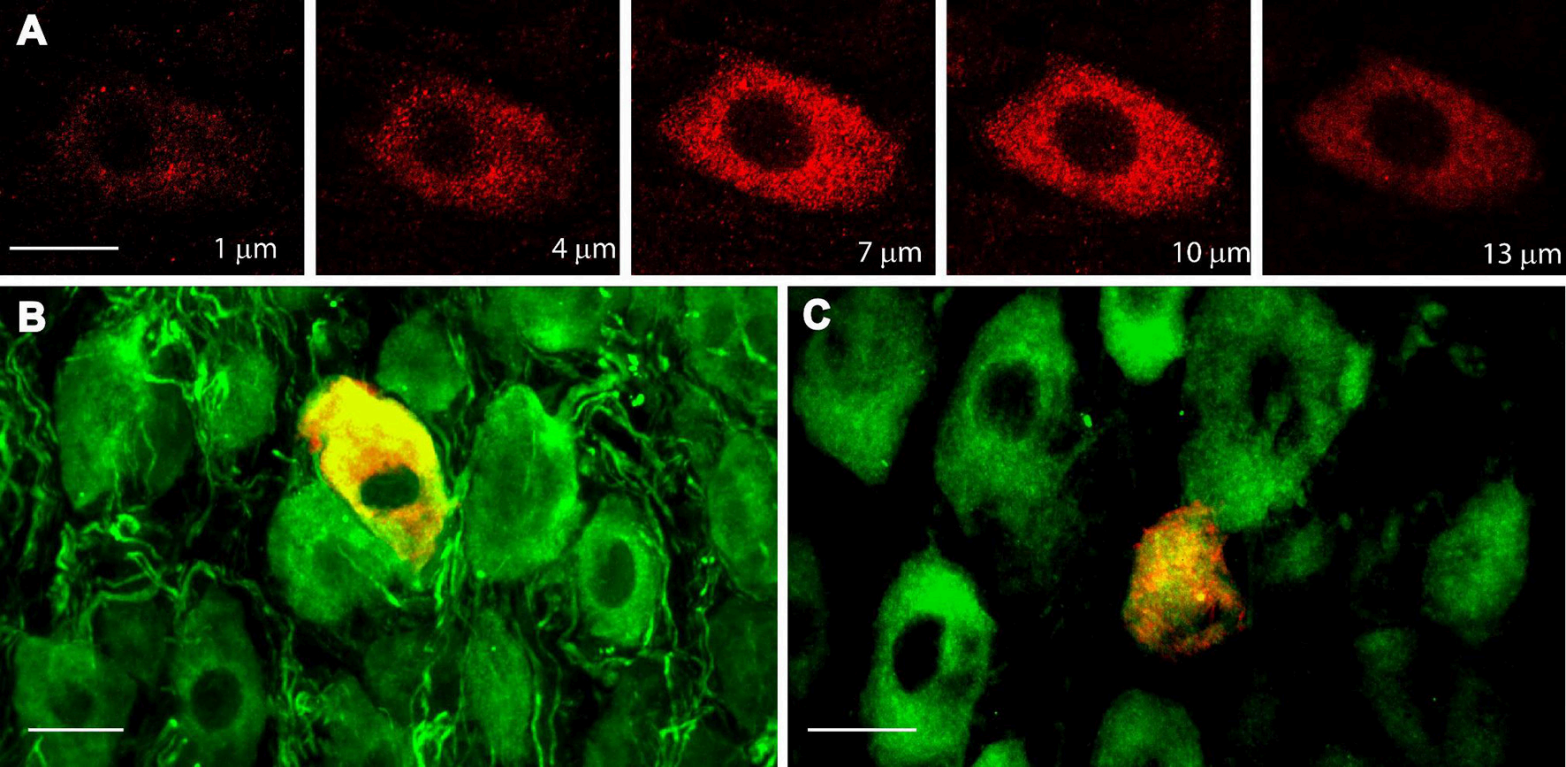
TrkB-Fl is non-uniformly expressed in the cytoplasm of ganglionic neurons, which co-express tyrosine hydroxylase or p75NTR. **(A)** TrkB immunolabeling (red) occurred in clusters throughout the cytoplasm of ganglionic neurons, as shown in these serial confocal optical 1-μm sections (z-axis) of SCG neurons. TrkB-Fl (red) was co-expressed with TH- **(B)** and p75NTR-IR neurons **(C)**. Scale bar = 10 μm.

In accordance with the mRNA expression levels found by Dixon and McKinnon (1994) and Wetmore and Olson (1995), an abundance of TrkA-IR and p75NTR-IR expression was detected in sympathetic neurons of adult SCG by immunohistochemistry (IHC; Figures 1A,B); the majority (82 ± 2%) of neurons expressed TrkA, while 51 ± 3% of neurons expressed p75NTR (Figure 1D). Most of the NtRs-IR was limited to the neuronal soma and was evenly distributed throughout the cytoplasm (Figure 2A). Interestingly, using an antibody directed against the catalytic intracellular domain, which is unique to the full-functional TrkB isoform, we detected a clear expression of TrkB-Fl in adult SCG neurons; indeed, 19 ± 2% of adult neurons were positive for TrkB-Fl (Figures 1C,D). An antibody directed against the extracellular domain of TrkB, which is present in both TrkB isoforms (full and truncated), revealed that 55 ± 4% of neurons were immunopositive. This result suggests that ~36% of ganglionic neurons would express only the truncated non-functional isoform of TrkB receptors (Figure 1D). Neurons positive for TrkB-Fl were scattered across the ganglia (Figure 1E). We also found that neurons positive for TrkB-Fl also co-expressed tyrosine hydroxylase (TH; Figure 2B), thus confirming their identity as sympathetic post-ganglionic neurons. It is known that p75NTR interacts with Trk receptors to regulate their affinity for neurotrophins (Esposito et al., 2001), therefore, we studied whether TrkB receptors co-localized with p75NTR, and found that some (~20%) of the p75NTR-positive neurons also expressed TrkB-Fl (Figure 2C).

### Expression of neurotrophin receptors is different in neonatal neurons

**Figure 3:**
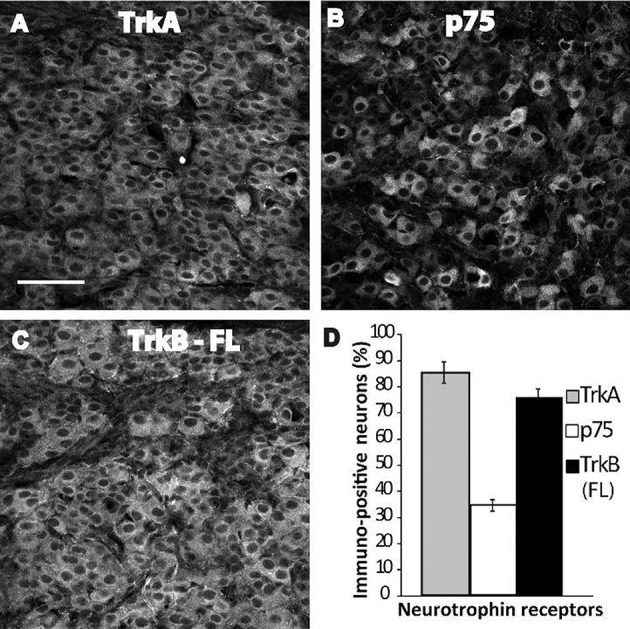
Abundant expression of TrkB-Fl in ganglia of neonatal rats. Examples of SCG neurons from early postnatal pups (day 4–6) immunolabeled against TrkA **(A)**, p75NTR **(B)**, and TrkB-Fl **(C)**. Sampling across the entire ganglion resulted in different proportions of immunopositive neurons; 86% of the neurons expressed TrkA, 35% expressed p75NTR, and 76% expressed TrkB-Fl **(D)**. Scale bar = 100 μm, *n* = 3.

The developmental regulation of Nt receptor mRNA is a well-known process; *trkA* mRNA, and particularly *p75* mRNA, both increase during early postnatal life in the rat SCG (Cowen et al., 2003). Similarly, *trkA* and *trkB* mRNAs increase by 7-fold and 2-fold, respectively, in the superior cervical ganglia between postnatal day 7 and adulthood (Ehrhard and Otten, 1994). Therefore, we characterized the expression of the Nts receptors in the SCG of neonatal rats by IHC to determine whether they were also developmentally regulated. In contrast to the findings obtained for adult neurons, TrkB-IR was abundantly expressed in sympathetic neurons in neonatal rats at P4–6 (Figure 3C), reaching similar expression levels as TrkA (Figure 3A). Of the neonatal neurons studied, 76 ± 3 and 86 ± 4% expressed TrkB and TrkA, respectively; whereas p75NTR-IR (Figure 3B) was expressed in only 35 ± 2% of neuronal soma (Figure 3D).

**Figure 4:**
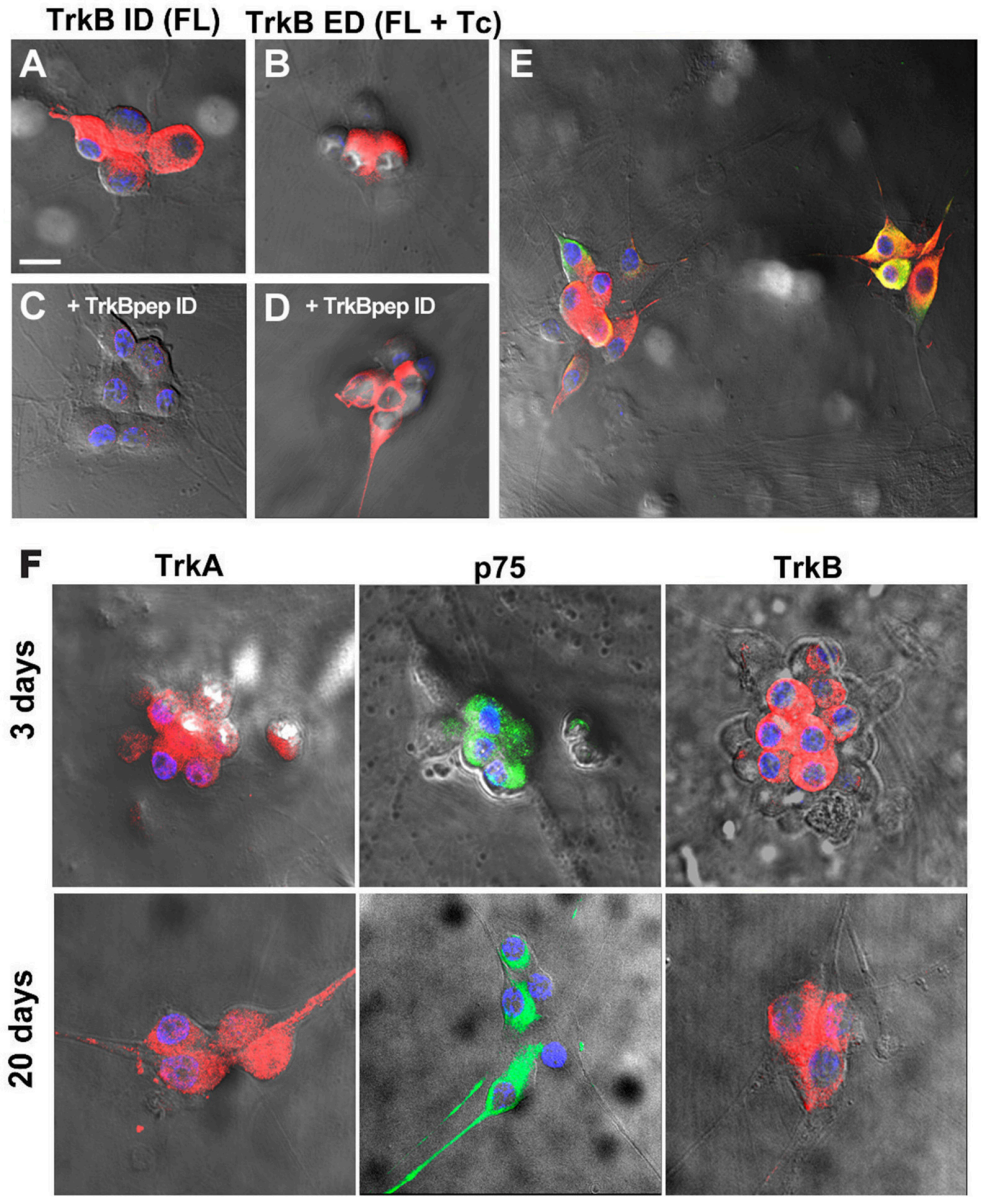
Ganglionic neurons of neonatal rats co-cultured with cardiomyocytes expressed TrkA, p75NTR, and TrkB-Fl. Neonatal co-cultured neurons immunolabeled for the intracellular domain of TrkB (specific for the TrkB-Fl isoform) **(A)** and for the extracellular domain (occurring in both TrkB isoforms) **(B)**. Pre-adsorption of the primary antibody against TrkB-ID with the corresponding peptide (1:10) removed labeling for the intracellular TrkB domain **(C)** but not for TrkB-ED **(D)**, thus confirming the specificity of the antibody. TrkB-Fl+ neurons were identified as sympathetic neurons through the co-expression of TH **(E)**. Scale bar = 30 μm. *n* = 4. Neurotrophin receptor expression is similar in SCG co-cultures in 3- and 20-days post culture preparations. Figure shows the expression of TrkA, p75NTR, and TrkB-Fl in SCG neurons at 3 DIV and 20 DIV **(F)**. *n* = 4. All cultures were counterstained with DAPI (blue). Scale bar = 25 μm. Supplementary material shows a 3D reconstruction of neurons immunostained with TrkB (red) and p75NTR (green) antibodies, and DAPI counterstaining (blue); video1 (merge), video2 (separated channels).

To determine whether the differential levels of Nts receptor expression found in neonatal neurons were preserved in cultured neurons, where functional studies have been performed, we characterized Nts receptor expression in sympathetic neurons co-cultured with cardiac myocytes at 3 DIV. The presence of TrkB was confirmed using the same two antibodies as described earlier, one directed against the intracellular catalytic domain present only in TrkB-Fl, and the other directed against the extracellular domain which recognizes both isoforms. High expression levels of TrkA and p75NTR were detected in cultured neurons (73 ± 3 and 75 ± 3%, respectively), as well as abundant expression of TrkB-IR (87 ± 4%), regardless of the antibody used (Figures 4A–D; Supplementary video). Most (~90%) of the TH-IR-positive neurons also expressed TrkB-Fl (Figure 4E). Since Luther and Birren (2006, 2009) reported that BDNF and NGF evoke time-dependent modulation of the electrophysiological properties of the neurons, we compared the expression pattern of TrkA, p75NTR and TrkB-Fl in 3 DIV and 20 DIV co-cultures of SCG, and found that the expression levels of the three receptors were maintained during this time (Figure 4F).

### Confirmation of the presence of TrkB-Fl in the adult SCG by western blotting and RT-PCR

**Figure 5:**
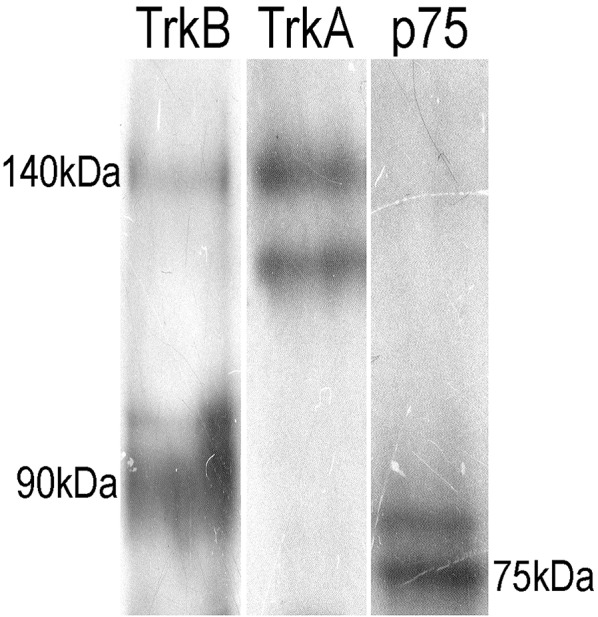
Immunoblotting of adult SCG proteins showing the presence of TrkB (two isoforms), p75NTR, and TrkA. Ganglia were homogenized in lysis buffer, proteins were separated by SDS-PAGE (12.5% resolving gel), and transferred onto a Immobilon-P transfer membrane. Western blot analysis was carried out for Nt receptors. The TrkB-ED antibody revealed the presence of the two isoforms of TrkB, a full-length 140 kD band and a truncated band of 90 kD. The other lanes show the presence of p75NTR (75 kD) and a doublet for TrkA.

**Figure 6:**
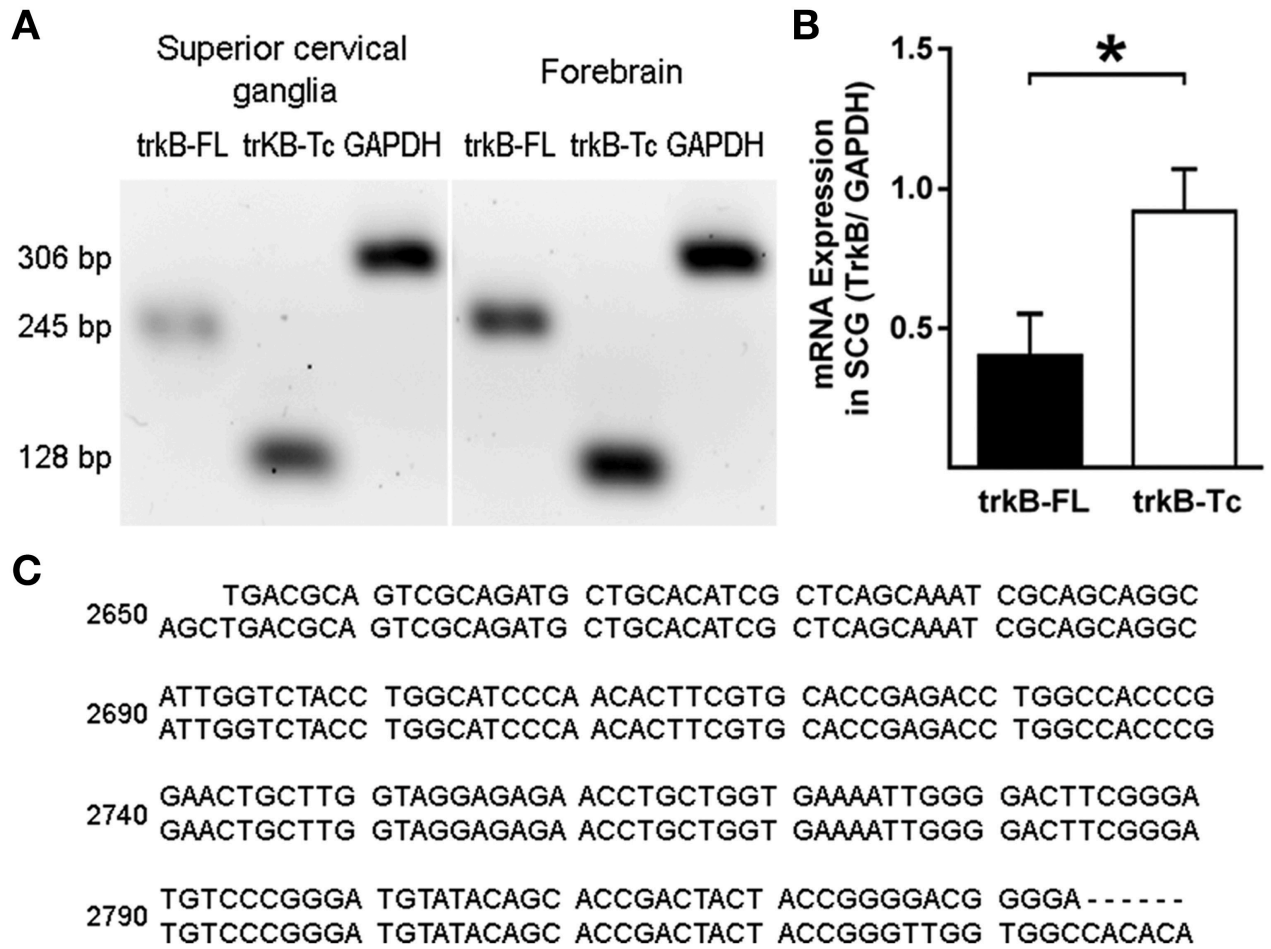
RT-PCR analysis of TrkB expression in the SCG and forebrain. Total mRNA was extracted from the SCG and forebrain (used as a control), and semi-quantitative RT-PCR was performed. **(A)** Expression of *trkB-Fl* and *trkB-Tc* in the SCG and forebrain. GADPH was used as a reaction control. **(B)** Graph showing differences in the expression of *TrkB-Tc* and *TrkB-Fl* mRNA; ^*^p < 0.05. **(C)** Sequences showing the alignment of RT-PCR products (upper lines) with the published GenBank sequence (https://www.ncbi.nlm.nih.gov/genbank/), confirming identity as *TrkB-Fl* (score 97–99%). *n* = 4.

To confirm the presence of the TrkB-Fl isoform in adult sympathetic SCG neurons, we performed Western blotting and RT-PCR analyses. Immunoblotting of SCG proteins with an anti-TrkB antibody directed against the extracellular domain (which recognizes both TrkB isoforms), revealed the presence of the two TrkB isoforms, a 140 kDa TrkB-Fl band and a 90 kDa TrkB-Tc band, with clearly stronger expression of the truncated isoform (Figure 5). The presence of TrkA and p75 receptors was revealed by staining with their respective antibodies (Figure 5). Regarding the TrkB transcript, it is known that the *trkB* gene is a complex transcriptional unit with multiple splicing forms ranging from 2 to 9 kb (Ninkina et al., 1996). We used primers directed against the two most common forms: *trkB-Fl* and *trkB-Tc1* (Kondo et al., 2010). Our RT-PCR analysis revealed that both transcripts were differentially expressed in the SCG (Figure 6A); *trkB-Tc* is 2.3-fold more abundant than the full isoform (*trkB-Fl*; Figure 6B). The expression of *trkB* in rat forebrain was used as a positive control (Figure 6A). The identity of the RT-PCR products was confirmed by sequence analysis, which matched the sequence published in the gene sequence bank for *trkB-Fl* (with 97–99% sequence identity; Figure 6C).

### BDNF and NGF induce the activation of Trk receptors

**Figure 7:**
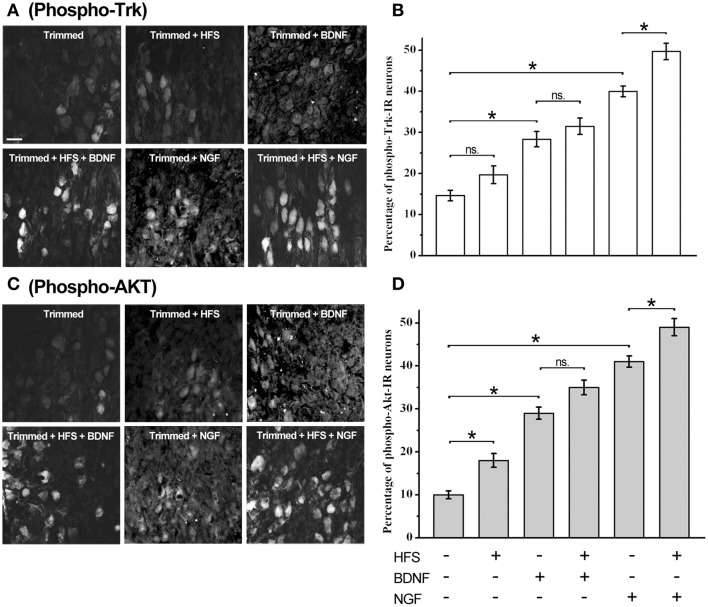
Trk receptors are activated by electrical stimulation, BDNF, and NGF in sympathetic neurons. Examples of sympathetic neurons from adult trimmed SCG immunolabeled against phospho-Trk **(A)** and phospho-Akt **(C)** under different experimental conditions. Scale bar = 50 μm. Bar graphs showing the averaged expression of phospho-Trk **(B)** and phospho-Akt **(D)** in the SCG, *n* = 4. Statistical analysis using one-way ANOVA and Sidak's multiple comparison test showed that high frequency stimulation (HFS) trended to increase the level of phospho-Trk+ (*p* > 0.05), but increased significantly the proportion of phospho-Akt+ cells from 10 ± 1 to 18 ± 2% (*p* < 0.005). HFS in combination with BDNF did not produce significant changes either in phospho-Trk+ or phospho-Akt+ cells (*p* > 0.05). In contrast, HFS in combination with NGF increased significantly the proportion of phospho-Trk+ and phospho-Akt+ cells, from 40 ± 1 to 50 ± 2% of phospho-Trk+ cells (*p* < 0.003), and from 41 ± 1 to 49 ± 2% of phospho-Akt+ cells (*p* < 0.004). Both neurotrophins increased significantly the proportion of phospho-Trk and phospho-Akt neurons. Thus, BDNF increased the proportion of phospho-Trk-IR and phospho-Akt+ neurons from 15 ± 1 to 29 ± 2% and from 10 ± 1 to 29 ± 1%, respectively (*p* < 0.0001). NGF was more efficient in the induction of Trk and Akt phosphorylation, thus, NGF induced Trk phosphorylation from 15 ± 1% to 40 ± 1% of cells (*p* < 0.0001) and the number of phospho-Akt+ cells from 10 ± 1 to 41 ± 1% (*p* < 0.0001). ^*^Significant differences between groups.

Our data clearly showed that *trkB-Fl* transcripts and protein are expressed in SCG, as well as TrkA and p75NTR. To address whether Trk receptors were functional in adult SCG, we trimmed the ganglion, to enhance its accessibility to Nts, and determined the phosphorylation state of Trk receptors and the downstream Trk-activated Akt pathway (Figure 7), in response to NGF and BDNF application, and in response to high frequency stimulation (HFS, 40 Hz-3 s). We compared the effect of each treatment by a one-way ANOVA test (Figures 7B,D). We found that trimming the ganglion increased significantly the proportion of phospho-Trk+ and phospho-Akt+ cells in relation to basal condition, 15 ± 1 vs. 5 ± 1% for phospho-Trk+ cells, and 10 ± 1 vs. 4 ± 1% for phospho-Akt+ cells (trimmed vs. basal condition; *p* < 0.02). HFS did not increase the level of phospho-Trk+ (*p* > 0.05), but increased significantly the proportion of phospho-Akt+ cells from 10 ± 1 to 18 ± 2% (*p* < 0.005). HFS in combination with BDNF did not produce changes in the proportion of phospho-Trk+ or phospho-Akt+ cells (*p* > 0.05). On the other hand, HFS in combination with NGF increased significantly the proportion of phospho-Trk+ and phospho-Akt+ cells, from 40 ± 1 to 50 ± 2% of phospho-Trk+ cells (*p* < 0.003) and from 41 ± 1 to 49 ± 2% of phospho-Akt+ cells (*p* < 0.004). NTs alone increased Trk and Akt phosphorylation, in comparison with trimmed ganglia, BDNF increased significantly the number of phospho-Trk-IR neurons, from 15 ± 1 to 29 ± 2% (*p* < 0.0001), and the number of phospho-Akt+ neurons, from 10 ± 1 to 29 ± 1%, (*p* < 0.0001).

Interestingly, NGF was more efficient in the induction of Trk and Akt phosphorylation, thus, NGF significantly increased Trk phosphorylation, in comparison with trimmed ganglia, from 15 ± 1 to 40 ± 1% of neurons (*p* < 0.0001), whereas Akt phosphorylation increased from 10 ± 1 to 41 ± 1% of neurons (*p* < 0.0001). These differences between NGF and BDNF were also observed when neurotrophins were applied with HFS. The difference between the effects of NGF and BDNF could be related to the differential expression of TrkA and TrkB receptors in adult sympathetic neurons.

## Discussion

Here, we present compelling evidence revealing that adult SCG neurons express functional TrkB-Fl receptors in addition to the known expression of TrkA and p75NTR receptors. This finding supports the idea that NGF and BDNF act upon TrkA and TrkB receptors, and despite having similar signaling pathways, may evoke different responses. The main points of our research can be summarized as follows: (1) The presence of TrkB-Fl+ neurons in the SCG was revealed by IHC, and the presence of TrkB protein and mRNA transcripts was confirmed by Western blotting and RT-PCR studies, respectively. Western-blot data showed clear evidence of 140 kDa and 90 kDa proteins, while RT-PCR revealed the presence of *trkB*-Fl along with *trkB-Tc* transcript products; the former was sequenced to confirm its identity; (2) This differential Nts receptor expression pattern was characterized in neonatal rat tissues and in neuronal culture; (3) Finally, we present evidence that electrical stimulation, exogenously applied NGF or BDNF, or a combination of both procedures, led to phosphorylation of the Trk receptor and the downstream Akt signaling pathway, indicating that both TrkA and TrkB receptors are present and functional.

Dixon and McKinnon (1994), and Wetmore and Olson (1995), using an RNase protection assay and an *in situ* hybridization assay, respectively, reported that adult SCG neurons expressed mRNAs encoding for *trkA* and *p75NTR*, as well as for a non-functional *trkB-Tc* isoform. Both groups concluded that functional *trkB-Fl* mRNA was not, or was barely, expressed in the SCG (Dixon and McKinnon, 1994; Wetmore and Olson, 1995). On the other hand, Ehrhard and Otten (1994), who, like us, used an RT-PCR approach, demonstrated not only the presence of both *trkB* mRNA isoforms in rat sympathetic ganglia, but also their increased expression during postnatal development. Since then, there have been no further reports investigating TrkB presence in SCG. In our previous research, we observed a differential effect of BDNF and NGF upon ganglionic LTP. Considering the known opposing effects of TrkB and TrkA activation (Prakash et al., 1996; Scott and Ramer, 2010), as well as the fact that some SCG neurons express the TrkB receptor (Arias et al., 2014), we hypothesized that some of the BDNF effects upon LTP could be mediated by the activation of TrkB receptors. To corroborate our hypothesis, we have now addressed whether the TrkB protein and the mRNA transcript are present in the SCG. Using three experimental approaches, our current research revealed that TrkB-Fl is indeed expressed in the SCG, but to a lesser extent than TrkB-Tc. Thus, immunohistochemical data clearly indicated the presence of TrkB-Fl in 19% of sympathetic ganglionic neurons, whereas, an antibody directed against an extracellular domain common to both TrkB-Fl and TrkB-Tc isoforms immunolabeled ~54% of the sympathetic neurons, suggesting that TrkB-Tc is present in ~35% of them (Figure 1). TrkB immunolabeling was detected in the soma of sympathetic neurons (Figure 2). Drake et al. (1999) also showed that TrkB-IR was localized as discrete clusters in axons, dendritic spines, and in the somata of neurons in the adult rat hippocampus (Drake et al., 1999). As expected, TrkA-IR was detected in the majority of neurons, whereas p75NTR was expressed in only 50% of neurons. Accordingly, it is reasonable to suppose that sympathetic neurons can express more than one type of Nt receptor, which would enrich their synaptic modulatory capability evoked by Nts. Using DAPI to distinguish neurons from Schwann cells, we are confident that only the neurons were immunolabeled and analyzed. In accordance with our immunohistochemical analyses, Western-blotting results showed a clear expression of a 140-kDa protein band and a more than 3-fold expression of a 90-kDa protein band, which match the reported electrophoretic profiles of TrkB-Fl and TrkB-Tc, respectively. Finally, RT-PCR also confirmed the presence of *trkB-Fl* and *trkB-Tc* transcripts, the latter was expressed 2.3-fold higher than *trkB-Fl* in adult SCG cells. Our data agree with those of Ehrhard and Otten (1994), but differ from Dixon and McKinnon (1994), and Wetmore and Olson (1995). It is possible that the different approaches used were responsible for these opposing results, since RT-PCR offers higher levels of specificity and sensitivity than traditional methods of RNA analysis. The significant TrkB-Tc expression identified suggests that this isoform could have a functional role in the SCG, beyond its role as a dominant-negative receptor which inhibits TrkB-Fl signaling, as reported in other systems (Baxter et al., 1997; Rose et al., 2003; Fenner, 2012). Collectively, these data clearly indicate that SCG neurons express the TrkB receptor in its full form. Consequently, the increase in LTP induced by BDNF (Arias et al., 2014), can be mediated not only by p75NTR, but also in part, by the activation of TrkB-Fl receptors.

Consistent with the known developmental regulation of Nt receptor expression (Ehrhard and Otten, 1994; Cowen et al., 2003), we observed different expression levels of Nt receptors in neonatal neurons compared with adult neurons. Thus, most of the neonatal neurons expressed TrkB-IR, reaching similar expression levels as TrkA (Figure 3); whereas p75NTR-IR was expressed in approximately one-third of neurons. Previous studies regarding the function of Nt receptors have been performed in neurons isolated from newborn animals and cultured with cardiac myocytes (Lockhart et al., 1997; Yang et al., 2002; Luther and Birren, 2009). The authors of these earlier studies concluded that BDNF activates p75NTR, whereas NGF activates either TrkA or p75NTR, to modify either neurotransmitter phenotype or sympathetic neuronal firing pattern (Yang et al., 2002; Luther and Birren, 2009; Luther et al., 2013). In these studies, the potential signaling role of TrkB-Fl upon sympathetic neuronal function was excluded as a possibility. Taking into account the high level of TrkB expression in neonatal neurons, we characterized whether its expression level was maintained in culture. Interestingly, we found that TrkA and TrkB were expressed at almost the same level (70–80%), whereas p75NTR was expressed in ~70% of neurons (Figure 4). We also investigated for evidence of temporal variation in the expression of Nt receptors, which could be correlated to the temporal neurotrophin modulation of the neuronal electrophysiological properties described by Luther and Birren (2006, 2009). However, we found that the three receptors were expressed at consistent levels when compared at 3 DIV and 20 DIV (Figure 4). Taking into account the high TrkB expression level detected in cultured neurons, it is reasonable to assume that at least some of the BDNF effects reported should be related to TrkB activation. Thus, it is very likely that the full-length TrkB isoform in the SCG has the functional capacity to activate specific signaling cascades and mediate effects upon synaptic transmission.

It is known that when receptor tyrosine kinases bind to their ligand, they dimerize, and this induces auto-phosphorylation and the activation of Ras/ERK1/2, PI3 kinase/Akt STAT, and phospholipase Cγ signaling pathways (Huang and Reichardt, 2003). Thus, we examined Trk receptor and Akt phosphorylation in SCG cells stimulated by NGF or BDNF, either alone, or in combination with electrical stimulation (Figure 7). Since BDNF and NGF are large and very basic proteins which do not readily penetrate tissues, we trimmed the ganglion to increase accessibility. Another issue to be considered is the presence of high affinity binding sites for the Nts, in other words their receptors, which precludes the diffusion of ligands to the inner part of the ganglion. However, our data clearly show that Trk receptors, and the downstream Akt signaling pathway, were phosphorylated by these treatments. From Figure 7, it is clear that the increase of p-Trk and p-Akt was dependent upon the application of neurotrophins, and that NGF was more effective than BDNF in the induction of p-Trk+ and p-Akt+ cells. This is consistent with the major presence of TrkA-IR cells (82%) relative to TrkB-IR (19%) cells in sympathetic ganglia (Figure 1). In response to BDNF, there were more p-Trk+ and p-Akt+ neurons (~30%) than BDNF-IR neurons (19%); this implies the existence of some form of interaction between the Trk receptors to reach this level of activation. Despite of the similarity between the signaling pathways activated by NGF and BDNF, it is reasonable to expect that the activation of either receptor would produce differential effects, such as the increase or decrease in LTP that we have observed. For instance, Scott and Ramer (2010) found that exogenous BDNF induces dendritic sprouting of deafferentated adult spinal cord neurons, whereas endogenous NGF severely restricted dendritic sprouting. These findings were replicated in p75NTR knockout mice supporting the idea that they were produced by TrkB and TrkA activation (Scott and Ramer, 2010). Similarly, Prakash et al. (1996) described rapid and opposite effects of BDNF and NGF upon the functional organization of the rat somato-sensory cortex (Prakash et al., 1996). Another degree of complexity arises with the signaling pathway that is preferentially activated. Barnabé-Heider and Miller (2003) demonstrated that BDNF and NT-3 acted upon TrkB and TrkC to promote the survival and neurogenesis of cortical progenitor cells. These authors also showed that neuronal survival depended upon the PI3-kinase pathway, whereas neurogenesis depended upon the MEK pathway (Barnabé-Heider and Miller, 2003). Furthermore, activation of the same Trk receptor by different ligands can evoke distinct responses. For example, NGF-TrkA signaling promotes neuronal survival in SCG neurons (Riccio et al., 1997), whereas NT-3, which also activates TrkA, cannot substitute for NGF in supporting survival from the distal axon (Kuruvilla et al., 2004).

In addition to the differential regulatory effects of NGF and BDNF upon LTP, our previous results showed evidence of a potential form of endogenous NGF-dependent LTP modulation (Arias et al., 2014). In the present study, we show additional evidence for this endogenous form of neurotrophin modulation, in that we found that trimming the ganglion, and electrical stimulation, without the application of Nts, still evoked a significant increase in p-Akt (Figure 7). A likely explanation for this is that both procedures release a minute amount of neurotrophin that would activate some Trk receptors, and in turn amplify this stimulus and lead to the increased activation of Akt, and resulting in a significant level of p-Akt expression. Gärtner and Staiger (2002) demonstrated that short high-frequency bursts of stimuli that induce LTP also evoke the instantaneous secretion of BDNF in primary cultures of hippocampal neurons. The fact that BDNF and NGF are expressed in the neurons of the SCG in adult rats (Wetmore and Olson, 1995; Vega et al., 2016) supports this possibility.

Data presented here clearly indicate that adult sympathetic neurons of the rat SCG express functional TrkB-Fl, along with TrkB-Tc, TrkA, and p75NTR. Therefore, some of the effects of BDNF in this ganglion should take into account the presence of this receptor. Considering the expression and co-expression of TrkA, p75NTR, and TrkB-Fl in adult SCG (Figures 1, 2), a likely interaction between these proteins is strongly expected. A similar interaction between p75 and TrkA has been reported. This interaction regulates the high affinity binding of NGF (Esposito et al., 2001), and supports the TrkA-promoted neuronal survival in hippocampal neurons (Culmsee et al., 2002). Therefore, the mechanisms involved in the neurotrophin-dependent modulation of LTP could involve the single or concomitant effects of one or more neurotrophins. We are currently investigating the contribution of ion channels and signaling pathways involved in this neurotrophin-dependent modulation of LTP.

## Author contributions

PV, MM, and FC conceived and designed the research, analyzed and interpreted results. PV, AC, BS, EA, DE, JF, and MZ performed the experiments. PV and BS prepared figures. PV drafted the manuscript. MM and FC edited and revised the manuscript. All authors approved the final version of the manuscript and agree to be accountable for all aspects of the work.

### Conflict of interest statement

The authors declare that the research was conducted in the absence of any commercial or financial relationships that could be construed as a potential conflict of interest. The reviewer MB and handling Editor declared their shared affiliation, and the handling Editor states that the process nevertheless met the standards of a fair and objective review.

## References

[B1] Alkadhi K. A. Otoom S. A. Tanner F. L. Sockwell D. Hogan Y. H. (2001). Inhibition of ganglionic long-term potentiation decreases blood pressure in spontaneously hypertensive rats. Exp. Biol. Med. 226, 1024–1030. 11743138 10.1177/153537020122601109

[B2] Arias E. R. Valle-Leija P. Morales M. A. Cifuentes F. (2014). Differential contribution of BDNF and NGF to long-term potentiation in the superior cervical ganglion of the rat. Neuropharmacology 81, 206–214. 10.1016/j.neuropharm.2014.02.001 24530966

[B3] Barnabé-Heider F. Miller F. D. (2003). Endogenously produced neurotrophins regulate survival and differentiation of cortical progenitors via distinct signaling pathways. J. Neurosci. 23, 5149–5160. 12832539 10.1523/JNEUROSCI.23-12-05149.2003 PMC6741181

[B4] Baxter G. T. Radeke M. J. Kuo R. C. Makrides V. Hinkle B. Hoang R.. (1997). Signal transduction mediated by the truncated trkB receptor isoforms, trkB.T1 and trkB.T2. J. Neurosci. 17, 2683–2690. 9092589 10.1523/JNEUROSCI.17-08-02683.1997 PMC6573096

[B5] Chao M. V. (2003). Neurotrophins and their receptors: a convergence point for many signalling pathways. Nat. Rev. Neurosci. 4, 299–309. 10.1038/nrn1078 12671646

[B6] Cifuentes F. Arias E. R. Morales M. A. (2013). Long-term potentiation in mammalian autonomic ganglia: an inclusive proposal of a calcium-dependent, trans-synaptic process. Brain Res. Bull. 97, 32–38. 10.1016/j.brainresbull.2013.05.011 23727546

[B7] Cowen T. Woodhoo A. Sullivan C. D. Jolly R. Crutcher K. A. Wyatt S.. (2003). Reduced age-related plasticity of neurotrophin receptor expression in selected sympathetic neurons of the rat. Aging Cell. 2, 59–69. 10.1046/j.1474-9728.2003.00035.x 12882335

[B8] Culmsee C. Gerling N. Lehmann M. Nikolova-Karakashian M. Prehn J. H. Mattson M. P.. (2002). Nerve growth factor survival signaling in cultured hippocampal neurons is mediated through TrkA and requires the common neurotrophin receptor p75. Neuroscience 115, 1089–1108. 10.1016/S0306-4522(02)00539-0 12453482

[B9] Dixon J. E. McKinnon D. (1994). Expression of the trk gene family of neurotrophin receptors in prevertebral sympathetic ganglia. Brain Res. Dev. Brain Res. 77, 177–182. 10.1016/0165-3806(94)90194-5 8174227

[B10] Drake C. T. Milner T. A. Patterson S. L. (1999). Ultrastructural localization of full-length trkB immunoreactivity in rat hippocampus suggests multiple roles in modulating activity-dependent synaptic plasticity. J. Neurosci. 19, 8009–8026. 10479701 10.1523/JNEUROSCI.19-18-08009.1999 PMC6782460

[B11] Ehrhard P. B. Otten U. (1994). Postnatal ontogeny of the neurotrophin receptors trk and trkB mRNA in rat sensory and sympathetic ganglia. Neurosci. Lett. 166, 207–210. 10.1016/0304-3940(94)90487-1 7513836

[B12] Elinos D. Rodríguez R. Martínez L. A. Zetina M. E. Cifuentes F. Morales M. A. (2016). Segregation of Acetylcholine and GABA in the rat superior cervical ganglia: functional correlation. Front. Cell. Neurosci. 10:91. 10.3389/fncel.2016.00091 27092054 PMC4823314

[B13] Esposito D. Patel P. Stephens R. M. Perez P. Chao M. V. Kaplan D. R.. (2001). The cytoplasmic and transmembrane domains of the p75 and Trk a receptors regulate high affinity binding to nerve growth factor. J. Biol. Chem. 276, 32687–32695. 10.1074/jbc.M011674200 11435417

[B14] Fenner B. M. (2012). Truncated TrkB: beyond a dominant negative receptor. Cytokine and growth factor. Reviews 23, 15–24. 10.1016/j.cytogfr.2012.01.002 22341689

[B15] Gärtner A. Staiger V. (2002). Neurotrophin secretion from hippocampal neurons evoked by long-term-potentiation-inducing electrical stimulation patterns. Proc. Natl. Acad. Sci. U.S.A. 99, 6386–6391. 10.1073/pnas.092129699 11983920 PMC122958

[B16] Gerges N. Z. Aleisa A. M. Alhaider A. A. Alkadhi K. A. (2002). Reduction of elevated arterial blood pressure in obese Zucker rats by inhibition of ganglionic long-term potentiation. Neuropharmacology 43, 1070–1076. 10.1016/S0028-3908(02)00283-6 12504912

[B17] Hawrot E. Patterson P. H. (1979). Long-term culture of dissociated sympathetic neurons. Meth. Enzymol. 58, 574–584. 10.1016/S0076-6879(79)58174-9 423793

[B18] Huang E. J. Reichardt L. F. (2003). Trk receptors: roles in neuronal signal transduction. Annu. Rev. Biochem. 72, 609–642. 10.1146/annurev.biochem.72.121801.161629 12676795

[B19] Kondo Y. Saruta J. To M. Shiiki N. Sato C. Tsukinoki K. (2010). Expression and role of the BDNF receptor-TrkB in rat adrenal gland under acute immobilization stress. Acta Histochem. Cytochem. 43, 139–147. 10.1267/ahc.10027 21245980 PMC3015051

[B20] Kuruvilla R. Zweifel L. S. Glebova N. O. Lonze B. E. Valdez G. Ye H.. (2004). A neurotrophin signaling cascade coordinates sympathetic neuron development through differential control of TrkA trafficking and retrograde signaling. Cell 118, 243–255. 10.1016/j.cell.2004.06.021 15260993

[B21] Laemmli U. K. (1970). Cleavage of structural proteins during the assembly of the head of bacteriophage T4. Nature 227, 680–685. 10.1038/227680a0 5432063

[B22] Lockhart S. T. Turrigiano G. G. Birren S. J. (1997). Nerve growth factor modulates synaptic transmission between sympathetic neurons and cardiac myocytes. J. Neurosci. 17, 9573–9582. 9391012 10.1523/JNEUROSCI.17-24-09573.1997 PMC6573427

[B23] Lu B. Figurov A. (1997). Role of neurotrophins in synapse development and plasticity. Rev. Neurosci. 8, 1–12. 10.1515/REVNEURO.1997.8.1.1 9402641

[B24] Lu L. Dempsey J. Liu S. Y. Bossert J. M. Shaham Y. (2004). A single infusion of brain-derived neurotrophic factor into the ventral tegmental area induces long-lasting potentiation of cocaine seeking after withdrawal. J. Neurosci. 24, 1604–1611. 10.1523/JNEUROSCI.5124-03.2004 14973246 PMC6730465

[B25] Luther J. A. Birren S. J. (2006). Nerve growth factor decreases potassium currents and alters repetitive firing in rat sympathetic neurons. J. Neurophysiol. 96, 946–958. 10.1152/jn.01078.2005 16707716

[B26] Luther J. A. Birren S. J. (2009). p75 and TrkA signaling regulates sympathetic neuronal firing patterns via differential modulation of voltage-gated currents. J. Neurosci. 29, 5411–5424. 10.1523/JNEUROSCI.3503-08.2009 19403809 PMC3326291

[B27] Luther J. A. Enes J. Birren S. J. (2013). Neurotrophins regulate cholinergic synaptic transmission in cultured rat sympathetic neurons through a p75-dependent mechanism. J. Neurophysiol. 109, 485–496. 10.1152/jn.00076.2011 23114219 PMC3545462

[B28] Ninkina N. Adu J. Fischer A. Piñón L. G. Buchman V. L. Davies A. M. (1996). Expression and function of TrkB variants in developing sensory neurons. EMBO. J. 15, 6385–6393. 8978665 PMC452462

[B29] Prakash N. Cohen-Cory S. Frostig R. D. (1996). Rapid and opposite effects of BDNF and NGF on the functional organization of the adult cortex *in vivo*. Nature 381, 702–706. 10.1038/381702a0 8649516

[B30] Riccio A. Pierchala B. A. Ciarallo C. L. Ginty D. D. (1997). An NGF-TrkA-mediated retrograde signal to transcription factor CREB in sympathetic neurons. Science 277, 1097–1100. 10.1126/science.277.5329.1097 9262478

[B31] Rose C. R. Blum R. Pichler B. Lepier A. Kafitz K. W. Konnerth A. (2003). Truncated TrkB-T1 mediates neurotrophin-evoked calcium signalling in glia cells. Nature 426, 74–78. 10.1038/nature01983 14603320

[B32] Scott A. L. Ramer M. S. (2010). Differential regulation of dendritic plasticity by neurotrophins following deafferentation of the adult spinal cord is independent of p75(NTR). Brain Res. 1323, 48–58. 10.1016/j.brainres.2010.02.004 20144886

[B33] Valle P. Cancino-Rodezno A. Sánchez M. Arias E. Elinos D. Feria J. (2014). Characterization of the Presence and Functionality of Neurotrophin Receptors in the Superior Cervical Ganglion of the Rat. Program No. 121.22. Neuroscience Meeting Planner. Washington, DC: Society for Neuroscience.

[B34] Vargas R. Cifuentes F. Morales M. A. (2007). Differential contribution of extracellular and intracellular calcium sources to basal transmission and long-term potentiation in the sympathetic ganglion of the rat. Dev. Neurobiol. 67, 589–602. 10.1002/dneu.20364 17443810

[B35] Vega A. Cancino-Rodezno A. Valle-Leija P. Sánchez-Tafolla B. M. Elinos D. Cifuentes F.. (2016). Neurotrophin-dependent plasticity of neurotransmitter segregation in the rat superior cervical ganglion *in vivo*. Dev. Neurobiol. 76, 832–846. 10.1002/dneu.22362 26562219

[B36] Wetmore C. Olson L. (1995). Neuronal and nonneuronal expression of neurotrophins and their receptors in sensory and sympathetic ganglia suggest new intercellular trophic interactions. J. Comp. Neurol. 353, 143–159. 10.1002/cne.903530113 7714245

[B37] Yan Q. Radeke M. J. Matheson C. R. Talvenheimo J. Welcher A. A. Feinstein S. C. (1997). Immunocytochemical localization of TrkB in the central nervous system of the adult rat. J. Comp. Neurol. 378, 135–157. 10.1002/(SICI)1096-9861(19970203)378:1<135::AID-CNE8>3.0.CO;2-5 9120052

[B38] Yang B. Slonimsky J. D. Birren S. J. (2002). A rapid switch in sympathetic neurotransmitter release properties mediated by the p75 receptor. Nat. Neurosci. 5, 539–545. 10.1038/nn0602-853 11992117

